# Pharmacokinetics and Disposition of Heparin-Binding Growth Factor Midkine Antisense Oligonucleotide Nanoliposomes in Experimental Animal Species and Prediction of Human Pharmacokinetics Using a Physiologically Based Pharmacokinetic Model

**DOI:** 10.3389/fphar.2021.769538

**Published:** 2021-11-03

**Authors:** Haihong Bai, Yuanguo Cheng, Jinjing Che

**Affiliations:** ^1^ Beijing Institute of Microbiology and Epidemiology, Beijing, China; ^2^ Phase I Clinical Trial Center, Beijing Shijitan Hospital, Capital Medical University, Beijing, China; ^3^ Beijing Institution of Pharmacology and Toxicology, Beijing, China

**Keywords:** hepatocellular carcinoma, antisense oligonucleotide drug, nanoliposome, pharmacokinetics, PBPK model

## Abstract

Encapsulating the antisense oligonucleotide drug MK-ASODN with nanoliposomes greatly improved its potency and targeting to the heparin-binding growth factor midkine. The disposition and pharmacokinetic (PK) parameters of MK-ASODN nanoliposomes were studied in monkeys and rats, and the human PK parameters were predicted based on preclinical data using a physiologically based pharmacokinetic (PBPK) model. Following intravenous injection, the drug plasma concentration rapidly declined in a multiexponential manner, and the drug was rapidly transferred to tissues from the circulation. The terminal t_1/2_ in plasma was clearly longer than that of the unmodified antisense nucleic acid drug. According to the AUC,MK-ASODN nanoliposomes were mainly distributed in the kidney, spleen, and liver. . MK-ASODN nanoliposomes were highly plasma protein bound, limiting their urinary excretion. Very little MK-ASODN nanoliposomes were detected in urine or feces. The plasma disposition of MK-ASODN nanoliposomes appeared nonlinear over the studied dose range of 11.5–46 mg kg^−1^. The monkey PBPK model of MK-ASODN nanoliposomes was well established and successfully extrapolated to predict MK-ASODN nanoliposome PK in humans. These disposition and PK data support further development in phase I clinical studies.

## 1 Introduction

Hepatocellular carcinoma (HCC), a primary malignancy of the liver, is one of the most frequently occurring tumors worldwide; however, no satisfactory therapeutic drugs or treatments are available (Dai et al., 2020; Qi et al., 2020). Recent insights into the biology of HCC suggest that certain growth factors and signaling factors overexpressed in tumor tissues are likely to play significant roles in the development of HCC by accelerating cell proliferation and invasion (Candia et al., 2020; Sathipati and Ho, 2020; Yim et al., 2020). These novel unexplored sites are expected to be potential targets for disease diagnosis and HCC drug development. Midkine (MK) is a heparin-binding growth factor that is highly expressed in HCC and other malignant tumors but is undetectable in most normal human tissues, such as the liver (Lu et al., 2020; Omran et al., 2020). MK mainly participates in tumor occurrence, antiapoptosis, migration and transformation through angiogenesis-mediated signal transduction, including protein tyrosine phosphatases, phosphatidylinositol 3-kinase, mitogen-activated protein kinase and extracellular regulated protein kinases (Filippou et al., 2020; Huang et al., 2015). Nevertheless, angiogenesis is the key to the unlimited invasion, growth and metastasis of HCC cells (Ma et al., 2018). Therefore, inhibiting the activity of MK is expected to be one of the most effective methods to treat HCC.

As a type of genetic therapy, antisense oligonucleotide (ASODN) drugs have greater specificity, superior efficacy, and lower toxicity than conventional drugs (Bennett., 2019; Gheibi-Hayat and Jamialahmadi, 2020). ASODNs constructed according to the sequence of mRNA encoding MK in primary HCC cells could specifically target MK to inhibit the growth of tumors in nude mice and other preclinical animal models (Dai et al., 2009; Murasugi, 2013). However, naked MK-ASODN can be rapidly degraded in the body and is polyanionic, both leading to a reduction in the amount of drug that can permeate into tumor cells. In addition, unmodified oligonucleotides tend to localize in endosomes/lysosomes, where they are unavailable for antisense purposes (Kuijper et al., 2021). To improve their cellular uptake and spatially and temporally controlled activity, MK-ASODN was packaged with nanoliposomes for stabilization and to enhance cell penetration (Zhong et al., 2013). The MK mRNA ASODN nanoliposomes targeting primary liver cancer cells in this study have independent intellectual property rights in China. The unique phospholipid bilayer of the nanoliposomes effectively protects the encapsulated oligonucleotides from degradation by enzymes or other active substances. Moreover, the surface charge characteristics of the nanoliposomes promote the affinity of MK-ASODN to most cell membranes. Both of these properties are especially beneficial for a prolonged duration of action, leading to enhanced efficacy compared with naked MK-ASODN. Moreover, because of the small size of the nanoliposomes, they localize to specific cells and locations in the reticuloendothelial system after entering systemic circulation, such as the liver, lung and bone marrow. Therefore, tissue targeting is greatly improved.

Currently, there have been few studies on the *in vivo* metabolic disposition of ASODN drugs, and obvious differences have been found compared with traditional small molecule chemical and macromolecular antibody drugs with respect to their *in vivo* behaviors (Geary et al., 2015).

Thus, preclinical pharmacokinetics (PK) and disposition of MK-ASODN nanoliposomes were evaluated in animals to support the associated safety and anticancer effect assessments performed in experimental animals and follow-up clinical trials. The PK and distribution parameters in rats and monkeys, as well as associated plasma protein binding studies, were performed to assess MK-ASODN nanoliposome absorption, distribution and elimination. The human PK parameters of the MK-ASODN nanoliposomes were then predicted using animal-to-human correlation analysis and physiologically based pharmacokinetic modeling (PBPK) simulations.

This is the first report showing the *in vivo* PK and distribution data of MK-ASODN nanoliposomes in rats and monkeys and the simulation and prediction of their human PK parameters. The results obtained in this study will help to guide dose selection for the first-in-human (FIH) study of MK-ASODN nanoliposomes and will provide useful information for the development of future ASODN nanoliposomes in this chemical class.

## 2 Materials and Methods

### 2.1 Chemicals and Materials

The MK-ASODN (5′-CCC​CGG​GCC​GCC​CTT​CTT​CA, 6044.4 Da) nanoliposomes (purity >99%) used in animal studies and a reference standard for analysis were obtained from the Huzhou Central Hospital (Zhejiang Province, China) (Zhong et al., 2013). Internal standard 5′CCTTGTTTCTACT, purity >98%, was synthesized in our laboratory. Human serum albumin, hexafluoroisopropanol (HFIP), triethylamine (TEA), acetonitrile and methanol were purchased from Sigma-Aldrich (St. Louis, MO, USA).

### 2.2 Experimental Animals

All animal care and use complied with the Guidance for Ethical Treatment of Laboratory Animals (The Ministry of Science and Technology of China, 2006; www.most.gov.cn/fggw/zfwj/zfwj2006). All animal studies were implemented according to the described protocols, which were reviewed and approved by the Institutional Animal Care and Use Committee at Beijing Xexbio Company (Beijing, China). Twenty Sprague-Dawley (SD) rats (male, weighing 200 ± 20 g) and nine macaques, five males and four females, each weighing approximately 6 kg, were provided by the Xieerxin Bioresource Institute, Beijing, China (license key SCXK (Jing)2005-0005, 2010-0007). The ethics number of the project is IACUC-XEX-0058. The animals were maintained at a controlled temperature (20–24°C) and relative humidity (40–70%) under a 12-h light/dark cycle. The animals were given commercial diets, except for an overnight fasting period before dosing, and filtered tap water ad libitum. The rats were acclimated to the facilities for 1 week before use and the monkeys were acclimated for 2 weeks.

### 2.3 Rat Studies

Rats were randomly assigned to four groups to receive a single intravenous (IV) dose of MK-ASODN nanoliposomes at 25 mg kg^−1^ (via the tail vein). Three groups of rats under isoflurane anesthesia were killed by bleeding from the abdominal aorta at 0.17, 0.5,2 and 6 h. Rat blood was collected in heparinized evacuated blood collection tubes and centrifuged to yield plasma fractions. The hearts, lungs, kidneys, jejunums, stomachs, fat, livers, spleens, and muscles were excised, rinsed in ice-cold saline, blotted, and weighed. For each gram of tissue, 10 ml of saline was added to make a tissue homogenate agitating the mixture in a homogenizer.

One group of rats were administered 25 mg kg^−1^ MK-ASODN nanoliposomes via the caudal vein and were housed individually in metabolic cages. Urine and fecal samples were collected from the rats before and from 0 to 4, 4–10, and 10–24 h after the single IV dose. The samples were weighed, and the urine collection tubes were frozen at −20°C. After collection of rat feces, a fecal slurry was made by adding 15 ml of an aqueous methanol solution (methanol:water = 50:50) to each gram of feces.

A liquid chromatography/tandem mass spectrometry (LC/MS/MS) method was used to analyze the MK-ASODN concentrations in the samples.

### 2.4 Macaque Monkey Study

Macaque monkeys were randomly assigned to one of three groups with 3 monkeys in each group. Each monkey received a single IV injection of MK-ASODN nanoliposomes at a dose of 11.5 mg kg^−1^, 23 mg kg^−1^, or 46 mg kg^−1^. Blood samples (approximately 0.5 ml) were collected in heparinized tubes and centrifuged to yield plasma fractions before drug administration and at 0.08, 0.17, 0.33, 0.5, 0.75, 1, 1.5, 2, 4, and 6 h after dosing. All monkey plasma samples were stored at −70°C pending analysis.

### 2.5 Plasma Protein Binding Assay

An ultrafiltration method was used to assess the whole plasma protein-binding characteristics of MK-ASODN nanoliposomes. Briefly, MK-ASODN nanoliposome quality control samples at high, medium, and low concentrations (0.25, 2.5, and 25 μg ml^−1^, respectively) were prepared in blank human plasma, human albumin, rat plasma, and monkey plasma.

The prepared samples were placed into a 4°C oven for 4 h. After sufficient binding of the drug to plasma proteins, 500 µl of each sample was placed in an ultrafiltration tube and centrifuged at 10,000 r min^−1^ for 25 min to determine the MK-ASODN concentration in both the ultrafiltrate and plasma compartments to calculate the plasma protein binding rate.

Formula(%fu)=(Concbufferchamber/Concplasmachamber)×100%

### 2.6 LC/MS-Based Bioanalytical Assays

An Applied Biosystems Sciex API 4000 Trap mass spectrometer (Toronto, Ontario, Canada) interfaced with a Turbo V ion source with an Agilent 1100 HPLC system separation module (USA) was used to analyze the MK-ASODN nanoliposomes in biological matrices. For MK-ASODN quantification in the various biological matrices, the mass spectrometry instrument parameters were optimized in positive ion mode to maximize the generation of MK-ASODN and internal standard protonated ions and to yield their characteristic product ions. The precursor-to-product ion pairs used for multiple reaction monitoring of MK-ASODN and the internal standard were m/z 695.8–304.2 and 675.8–319.0, respectively. For the quantification of MK-ASODN in biological matrices, the samples were extracted with phenol/dichloromethane (1:1, w/v). After centrifugation, the phenol/dichloromethane extracts were evaporated to dryness under a stream of N_2_ gas, and the resulting residues were reconstituted in the HPLC mobile phase before analysis. Chromatographic separation was achieved on a Waters XTerra^®^ MS C18 column (2.1 mm × 50 mm). The mobile phase consisted of 200 mM HFIP/2.85 mM TEA in water (solvent A) and 200 mM HFIP/2.85 mM TEA in 60:40 (v:v) methanol/water (solvent B). For quantification, the following gradient program was used: 5–100% solvent B over 3.0 min; 100% solvent B maintained for 4.0 min; and 100–5% solvent B over 0.5 min. The separation system was equilibrated with 5% solvent B for 4.5 min before the next analysis.

Matrix-matched calibration curves were constructed using weighted (1/X^2^) linear regression of the ratio of the peak areas of MK-ASODN to the internal standard (Y) versus the corresponding nominal MK-ASODN concentration (X, μg·ml^−1^). The assays were validated according to the FDA guidelines on bioanalytical validation (https://www.fda.gov/regulatory-information/search-fda-guidance-documents/bioanalytical-method-validation-guidance-industry) to demonstrate their reliability and reproducibility for the intended use.

### 2.7 PK Modeling and the Human PK Prediction Model

The MK-ASODN nanoliposome PBPK model was built by GastroPlus version 8.0 (Simulations Plus, Inc., Lancaster, CA, USA). In summary, this model was composed of 14 tissue compartments, including the heart, lung, brain, adipose tissue, muscle, skin, spleen, reproductive tissue, gastrointestinal tract, liver, kidney, yellow marrow, red marrow and the rest of the body. These compartments were linked together by venous and arterial blood circulation. All of the tissues were considered to be well-stirred compartments, and drug distribution into these compartments was driven by perfusion-limited kinetics. Each compartment was defined by an associated tissue blood flow rate, volume and a tissue-to-plasma partition coefficient (Kp). The Kp values were predicted using established tissue composition-based models (Rodgers et al., 2005; Rodgers and Rowland, 2006; Poirier et al., 2009), and they depended on compound biopharmaceutical properties.

In the monkey PBPK model, because of the specificity of nucleic acid liposome drugs, the main input parameters for the simulation were molecular weight, physiological parameters (Fup%) and monkey *in vivo* clearance (CL) values. The Kp values were predicted from the physicochemical properties as well as *in vitro* data inputs. Different built-in models/modules in GastroPlus^TM^ regarding the Kp predictions were compared to obtain a final PBPK model that best described the observed *in vivo* plasma–concentration profiles in monkeys, and the Lukacoval (Rodgers-Single) method was selected. The predicted steady-state volume (Vss) values from these Kps were closer to the results of the *in vivo* studies when Lukacoval’s (Rodgers-Single) method was used compared with the use of other methods. Thus, a PBPK model was built for monkeys, and the software defaulted to the monkey physiologic parameters, the above physicochemical properties and the MK-ASODN nanoliposome plasma concentration data from monkeys.

The simulated plasma concentration–time profile in monkeys was graphically coplotted with the observed PK data to evaluate the fidelity of the PK prediction by a full PBPK model. After optimizing the models in preclinical species, all of the selected built-in models/modules used in the animal PBPK model were used with humans to build a human PBPK model (Chinese, male). Then, the human Fup% were substituted into the model. The CL in humans was extrapolated by single-species allometric scaling from monkeys using the following equation:

$$Cl_{\mathrm{human}}=Cl_{\mathrm{animal}}{\left(\frac{BW_{\mathrm{human}}}{BW_{\mathrm{animal}}}\right)}^{\begin{array}{c} .\\ 0.8 \end{array}}$$

where BW is the body weight (kg).

Lukacoval’s (Rodgers-Single) method was also used to predict the Vss in humans. The monkey dose was 46 mg kg^−1^. According to the Conversion of Animal Doses to Human Equivalent Doses Based on Body Surface Area (Food and Drug Administration, 2005), the safety factor was 10, giving a human dose of 90 mg. Thus, the MK-ASODN nanoliposome concentration in human plasma could be predicted based on the established model.

### 2.8 Data Processing

The plasma PK parameters of the MK-ASODN nanoliposomes were determined using noncompartmental model analysis with the WinNonlin software (version 8.3). Excel was used to calculate other relevant statistical parameters.

## 3 Results

### 3.1 PK Data After IV Injection of MK-ASODN Nanoliposomes to Macaques

**FIGURE 1:**
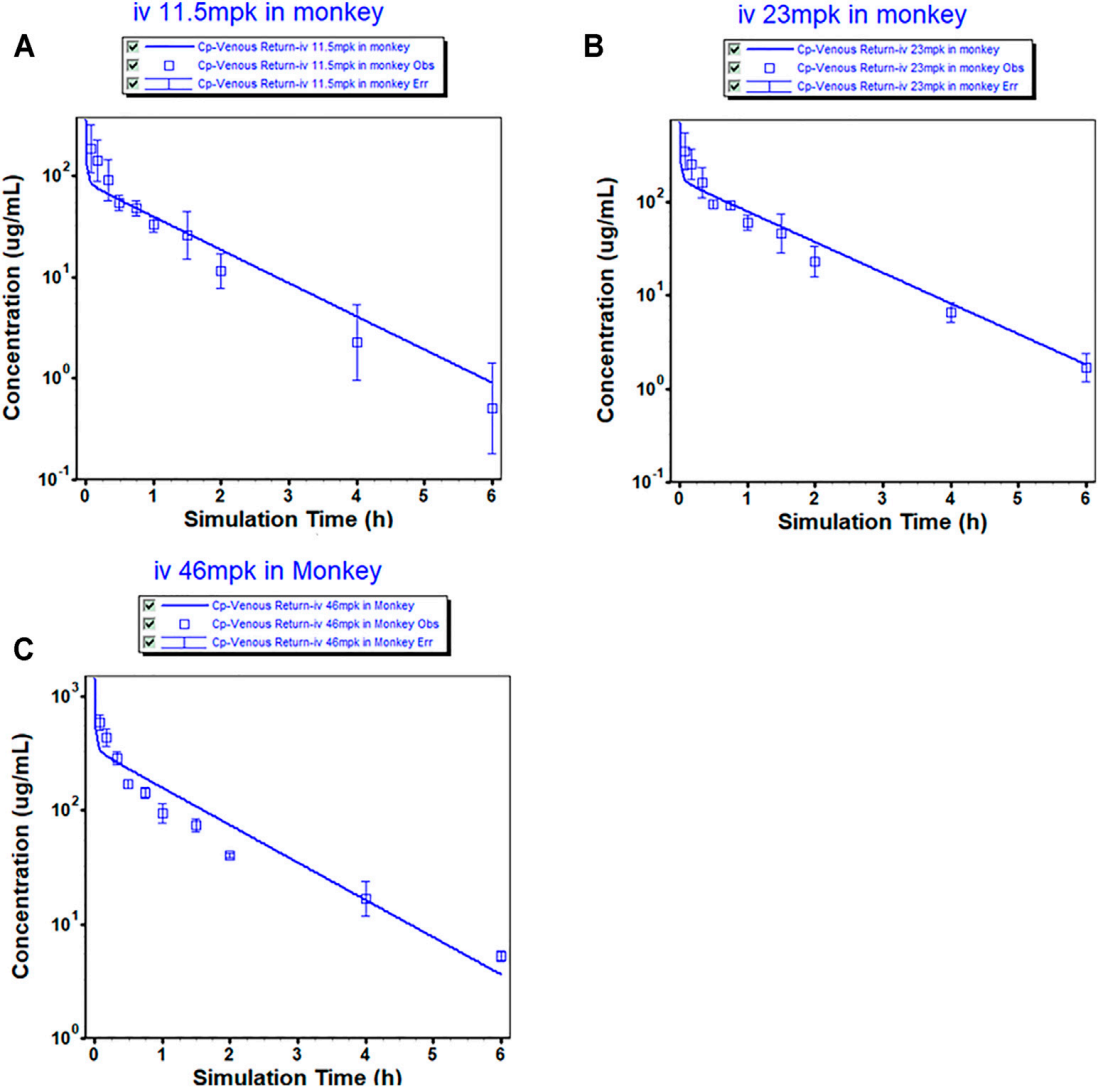
Observed (mean ± SD) and physiologically based pharmacokinetic (PBPK) model-simulated plasma concentration-time profile in monkeys after intravenous injection of MK-ASODN nanoliposomes at doses of 11.5, 23 and 46 mg kg^−1^ (*n* = 3).

The mean plasma concentrations of the MK-ASODN nanoliposomes over time after administration of a single dose to monkeys are shown in Figure 1 and the plasma PK parameters of the MK-ASODN nanoliposomes are summarized in Table 1.

**TABLE 1:** Pharmacokinetic parameters of MK-ASODN nanoliposomes following intravenous administration at doses of 11.5, 23 and 46 mg kg^−1^ to macaque monkeys (*n* = 3).

Pharmacokinetic parameter	11.5 mg kg^−1^	23 mg kg^−1^	46 mg kg^−1^
AUC(0-inf) (μg·h·ml^−1^)	122.04 ± 17.27	232.87 ± 18.14	415.38 ± 22.21
Cmax (μg·ml^−1^)	187.16 ± 102.84	347.43 ± 156.01	588.73 ± 88.88
MRT (h)	0.90 ± 0.45	1.04 ± 0.26	1.23 ± 0.16
CL (ml·kg^−1^·h^−1^)	95.41 ± 12.53	99.16 ± 7.64	110.95 ± 5.91
Vss (ml·kg^−1^)	88.41 ± 51.87	104.63 ± 34.41	137.89 ± 25.53
t1/2 (h)	0.79 ± 0.21	1.08 ± 0.18	1.33 ± 0.05

The macaques were IV administered three doses of MK-ASODN nanoliposomes (11.5 mg kg^−1^, 23 mg kg^−1^, and 46 mg kg^−1^), and the peak concentration (Cmax) values wery. All three de 187 ± 103, 347 ± 156, and 589 ± 88.9 μg ml^−1^, respectiveloses were detectable for up to 6 h after administration. The area under the curve from zero to infinity (AUCinf) values were 122.04 ± 17.27, 232.87 ± 18.14, and 415.38 ± 22.21 μg h ml^−1^, respectively, and dose linearity was assessed as a nonlinear PK characteristic (Table 2).

**TABLE 2:** Summary of statistical analysis for dose-proportionality for 1.5, 23 and 46 mg kg^−1^ MK-ASODN nanoliposomes in macaque monkeys.

PK parameter	unit	Predicted geometric mean	Slope estimate (90% CI)	Rdmn (90%CI)	Conclusion
AUC(0-inf)	μg·h·ml^−1^	(123.19, 423.10)	0.89 (0.79, 0.99)	0.86 (0.75,0.99)	Inconclusive
Cmax	μg·ml^−1^	(165.17, 591.32)	0.92 (0.41, 1.44)	0.90 (0.44, 1.84)	Inconclusive

According to the noncompartmental model analysis, the mean residence time (MRT) values were 0.90 ± 0.45, 1.04 ± 0.26, and 1.23 ± 0.16 h, respectively; the CL values were 95.41 ± 12.53, 99.16 ± 7.64, and 110.95 ± 5.91 ml kg^−1^·h^−1^, respectively; and the terminal half-lives of the elimination phase (t_1/2_) of the three doses were 0.79 ± 0.21, 1.08 ± 0.18, 1.33 ± 0.05 h, respectively. There was a statistically significant difference (*p* < 0.05) of the t_1/2_ between the low and medium dose groups, although there was no statistically significant difference (*p* > 0.05) in the t_1/2_ values of the medium and high dose groups. Therefore, the t_1/2_ was dose-dependent. The terminal t_1/2_ in plasma was clearly longer than that of the unmodified antisense nucleic acid drug. The V_ss_ values of the three doses were 88.41 ± 51.87, 104.63 ± 34.41, and 137.89 ± 25.53 ml kg^−1^, respectively, indicating that the MK-ASODN nanoliposomes were distributed in large quantities to tissues.

### 3.2 Tissue Distribution and Excretion of the MK-ASODN Nanoliposomes After IV Injection to SD Rats

**FIGURE 2:**
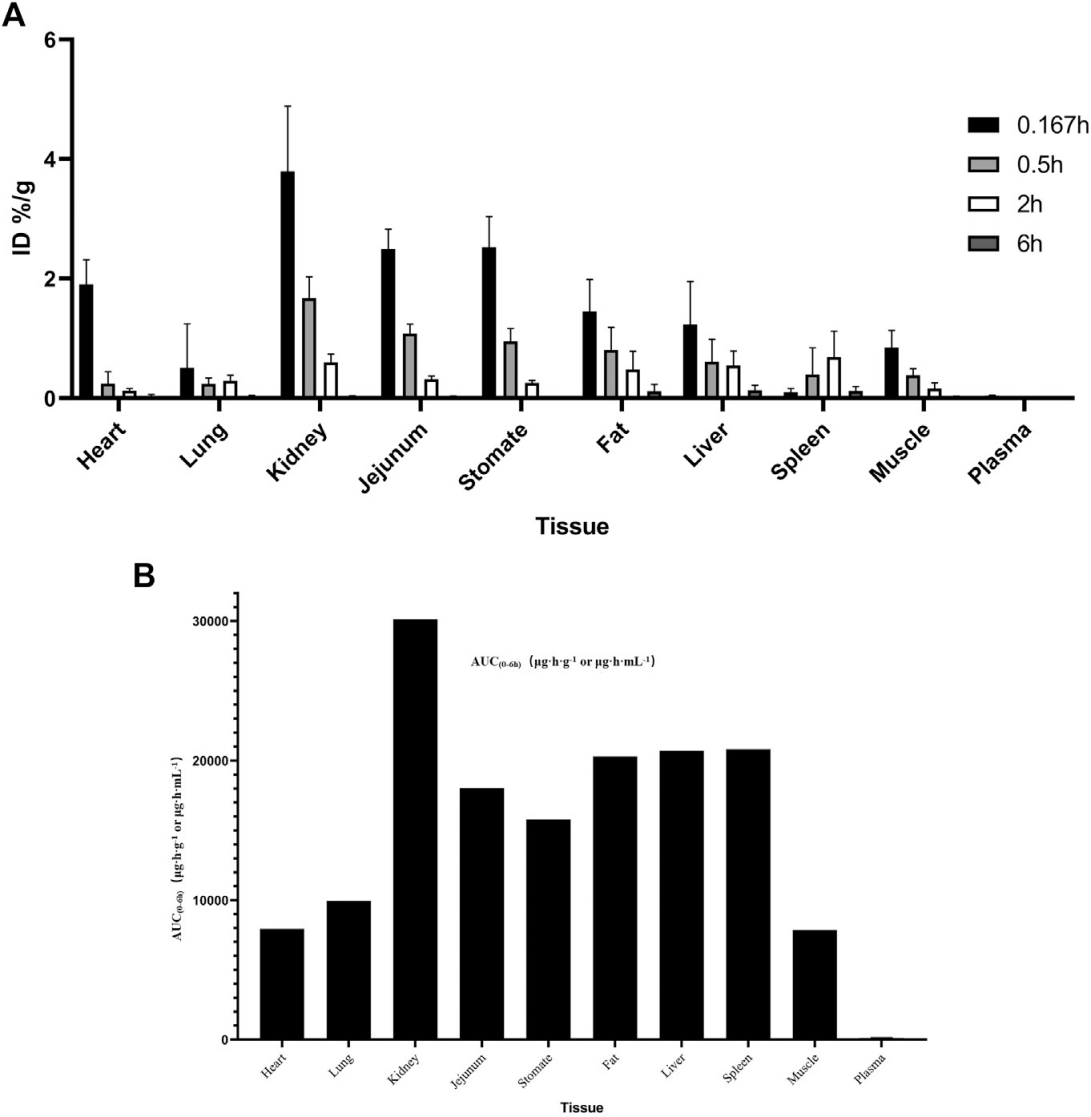
Tissue distribution of MK-ASODN nanoliposomes following intravenous administration at a dose of 25 mg·kg^−1^ to rats (*n* = 5). **(A)** ID%/g of tissues, **(B)** AUC of tissues.

The distribution of the MK-ASODN nanoliposomes in the major tissues after 25 mg kg^−1^ tail vein injection to SD rats is shown in Figure 2. Levels of various tissue exposures to MK-ASODN in rats were significantly higher than the associated systemic exposure level (Figure 2A). And MK-ASODN nanoliposomes were ranked by their area under the curve (AUC) values from smallest to largest in the following order: plasma, muscle, heart, lung, stomach, jejunum, fat, liver, spleen, kidney (Figure 2B). Thus, the MK-ASODN nanoliposomes were mainly distributed in the kidney, spleen, and liver.

### 3.3 Fecal Urinary Excretion of the MK-ASODN Nanoliposomes After IV Injection to SD Rats

No MK-ASODN prototype drug was detected in the urine or fecal plasma after 25 mg kg^−1^ tail vein injection in SD rats.

### 3.4 Plasma Protein Binding Rate

The binding of MK-ASODN nanoliposomes to plasma proteins from human plasma, human serum albumin, rat plasma, and monkey plasma ranged from 92 to 99%, and the binding of the MK-ASODN nanoliposomes to human plasma proteins was slightly higher than that to human serum albumin, indicating that the ASODNs may bind to other proteins in addition to albumin, e.g., α1-acid glycoproteins and lipoproteins. The results of the plasma protein binding rates are listed in Table 3.

**TABLE 3:** Plasma protein binding rates *in vitro* and in Rat, Monkey, and human plasma (*n* = 3).

	Nominal concentration (μg·ml^−1^)		
	0.25	2.5	25
	μg·ml^−1^	μg·ml^−1^	μg·ml^−1^
Human Albumin	92 ± 2.0	91 ± 4.4	93 ± 2.6
Human Plasma	96 ± 2.3	99 ± 0.2	99 ± 0.1
Rat Plasma	95.8 ± 2.5	97 ± 0.2	97 ± 0.2
Monkey Plasma	94 ± 7.2	97 ± 0.3	95 ± 3.3

### 3.5 Bioanalytical Method

The linear range of the LC/MS/MS method for detecting MK-ASODN in macaque plasma, tissue, feces and urine samples was 0.18–200 μg ml^−1^. The intrarun accuracy and precision in plasma were 95.9∼101% and 4.42∼8.99%, respectively, and the interrun accuracy and precision in plasma were 97.2∼103% and 6.36∼12.3%, respectively. The intrarun accuracy and precision in tissue were 98.1∼99.1 and 3.55% ∼ 5.12%, the interrun accuracy and precision in tissue were 99.2∼101.3 and 4.28% ∼ 7.26%, the intrarun accuracy and precision in urine were 99.1∼104.6% and 4.45∼5.09%, and the interrun accuracy and precision in urine were 99.7 ∼ 102.3% and 5.23 ∼ 8.00%, respectively. These data show that the stability of the method was good. The established bioanalytical method meets the needs to determine the PK parameters and tissue distribution of MK-ASODN nanoliposomes.

### 3.6 PK Modeling and the Human PK Prediction Model

The monkey PBPK model was used to construct a plasma concentration-time curve (Figure 1). The predicted and observed PK parameters with their corresponding prediction accuracy values are summarized in Table 4. The simulated IV plasma concentration-time profile of the MK-ASODN nanoliposomes from the PBPK model corresponded well with the observed profile. Most of the predicted PK parameters were reasonably consistent (<2-fold error) with the observed values. Therefore, the monkey PBPK model was established.

**TABLE 4:** Observed and simulated pharmacokinetic parameters of MK-ASODN nanoliposomes after intravenous administration to monkeys and humans.

Dose	Data source	Cmax (μg·ml^−1^)	AUC0–inf (μg·h·ml^−1^)
Monkey	Observed	187.16	122.04
11.5 mg/kg	Predicted	84.09	114.77
	Fold of error	2.22	0.94
Monkey	Observed	347.43	232.87
23 mg/kg	Predicted	172	229.54
	Fold of error	2.00	0.98
Monkey	Observed	588.73	415.38
46 mg/kg	Predicted	341	459.09
	Fold of error	1.73	1.10
Human 90 mg	Predicted	49.98	22.48

**FIGURE 3:**
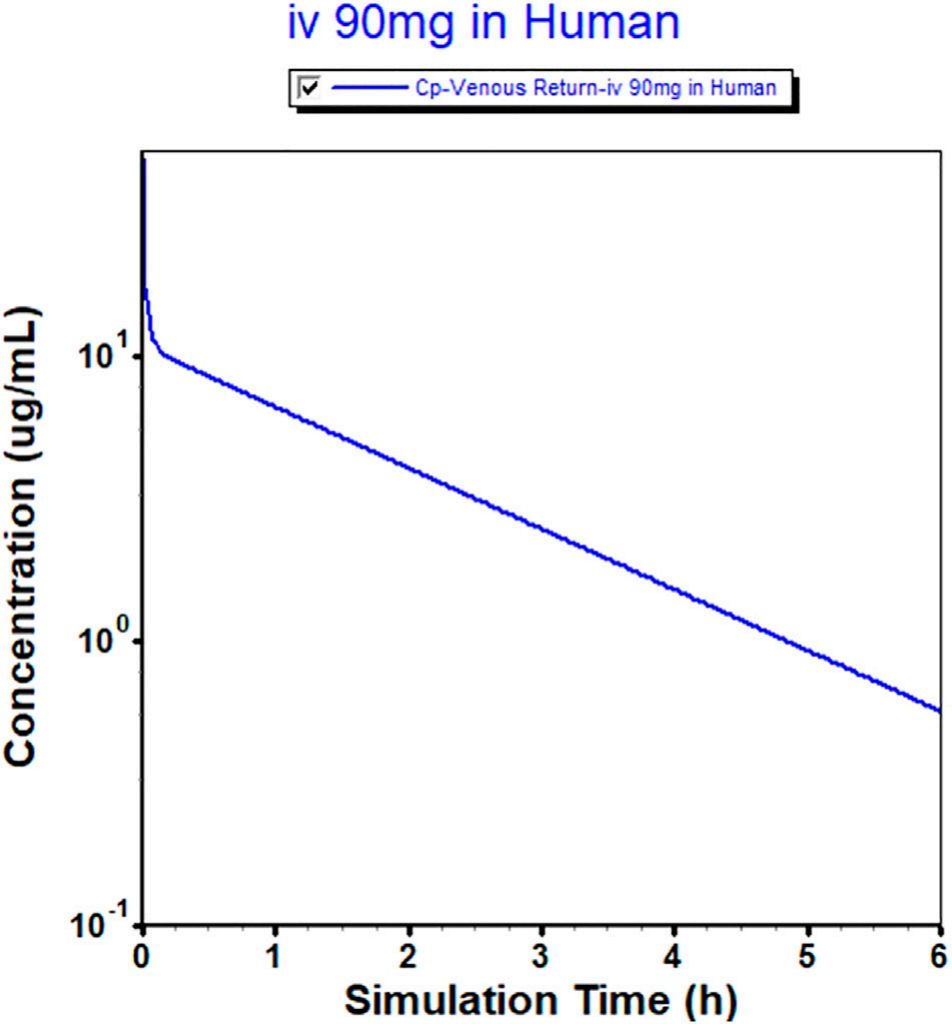
Physiologically based pharmacokinetic (PBPK) model-simulated plasma concentration-time profile in humans after intravenous injection of 90 mg of MK-ASODN nanoliposomes.

The successfully established monkey PBPK model was then extrapolated to humans, and a plasma concentration versus time profile was created for humans. The human CL, extrapolated from monkeys, was 4 L h^−1^, and the Vss was 7.89 L. The predicted plasma concentration-time curves are shown in Figures 1, 3. The predicted PK parameters with their corresponding prediction accuracy values are summarized in Table 4.

## 4 Discussion

MK-ASODN nanoliposomes are an antisense nucleic acid liposomal drug that targets liver tumor cells. However, natural antisense nucleic acid drugs are rapidly cleared from the circulatory system via renal and hepatocyte cell surface elimination receptors (Stanley et al., 2016). Therefore, to successfully develop antisense nucleic acid drugs, the barriers of cellular uptake and endosomal escape, prevention of nuclease-mediated degradation, and inhibition of immune activation need to be overcome to extend the presence of the unmodified nucleotides in systemic circulation from seconds or minutes to hours. Therefore, antisense nucleic acids must be appropriately chemically modified or protected to become druggable (Ochoa and Milam., 2020). The unique phospholipid bilayer of nanoliposomes effectively protects the encapsulated oligonucleotides from degradation by enzymes or other active substances.

Our experimental results demonstrated that MK-ASODN nanoliposomes exhibited multicompartmental nonlinear PK characteristics, which are mainly caused by the combination of transmembrane transport into cells, high hepatorenal accumulation, a low metabolic rate and slow drug release from the tissues back to circulation. The terminal t_1/2_ in plasma was clearly longer than that of the unmodified antisense nucleic acid drug.

MK-ASODN nanoliposomes were cleared from the plasma with a MRT of approximately 1 h. This more rapid plasma clearance may be attributed to efficient tissue uptake that primarily occurred in hepatocytes. Plasma clearance was dose-dependent and more rapid at low doses, suggesting a faster and greater distribution/uptake into the liver.

The tissue distribution results indicated that the MK-ASODN nanoliposome drug was rapidly distributed into tissues from plasma, resulting in several hundred-fold higher exposure to tissue than plasma. Additionally, MK-ASODN nanoliposome tissue uptake was heterogeneous. According to the AUC,MK-ASODN nanoliposomes were mainly distributed in the kidney, spleen, and liver. The liver is one of the main organs where MK-ASODN nanoliposomes are distributed in the body, providing experimental evidence for MK-ASODN nanoliposomes as a therapeutic agent targeted to the liver.

After intravenous administration, nanoliposomes are opsonized in the bloodstream before being phagocytized by macrophages and accumulated in the RES organs. This passive targeting promotes the accumulation of the nanoliposomes in the liver, a process that increases within tumors due to the EPR (enhanced permeability and retention) effect. Nanoliposomes improve their poor penetration into cells, allowing them targeting to Kupffer cell. *In vivo* studies in mice demonstrate that, at the level of Kupffer cells, the cellular uptake of nanoparticles is produced via mechanisms of phagocytosis and clathrin- and caveolin-mediated endocytosis and their release through the lysosomal and multivesicular pathways. Kupffer cells internalize nanoliposomes through multiple scavenger, toll-like, mannose, and Fc receptors. Nanoliposomes can be decorated for active vectorization with surface modifiers, that has been proposed for the treatment of liver cancer cells in order to increase nanoliposomes uptake by macrophages via receptor-mediated endocytosis. In summary, preferentially uptake of nanoliposomes by liver macrophages make them suitable vehicles for the vectorization of drugs for the treatment of liver cancer cells (Colino et al., 2020).

MK-ASODN nanoliposomes bind extensively to plasma proteins, mostly albumin, with plasma protein binding rates approximated across species. A higher plasma protein binding rate guarantees a slow elimination rate, a long duration of action and maintenance of MK-ASODN nanoliposomes *in vivo*. MK-ASODN nanoliposomes were highly bound to plasma proteins, which limited glomerular filtration and urinary excretion.

Very little MK-ASODN nanoliposomes were detected in urine or fecal in the form of the prototype drug. It has been suggested that ^3^H-labeled MK-ASODN isotope assays could be used to examine the fecal and urinary excretion of metabolites (Li et al., 2020). The low urinary excretion rate of the MK-ASODN nanoliposomes was consistent with tissue uptake being the primary mechanism of plasma clearance. The primary route of elimination of MK-ASODN nanoliposomes was found to be nuclease-mediated metabolism in tissues. Once generated, these chain-shortened metabolites were rapidly eliminated in urine due to their reduced binding to tissues and plasma proteins (Geary et al., 2009; Kaczmarkiewicz et al., 2019). Because nuclease metabolism is the rate-limiting step, no smaller metabolites accumulate within the tissues or plasma. Additionally, because of their reduced binding to plasma proteins, the shortened endonucleolytic products were rapidly eliminated in the urine (Geary et al., 2003; Yu et al., 2007).

To improve and expedite clinical drug candidate selection, there has been an increased demand to predict the PK parameters in humans as early as possible during development (Arya and Venkatakrishnan, 2020). Model reliability and predictability are crucial for this purpose (Rostami-Hodjegan and Tucker, 2007; Rostami-Hodjegan, 2012). Therefore, a full PBPK model was used for all simulations, in both preclinical species and humans, with the advanced compartmental absorption and transit (ACAT) model (Yu and Amidon, 1999; Agoram et al., 2001). When evaluating the nonclinical safety of phosphatidyl antisense drugs, monkeys are considered the most appropriate animal species because the dose-limiting toxic reactions (e.g., complement activation) that occur in monkeys are the same as those in humans but not in rodents.

CL is one of the most important drug disposition parameters and can be generally predicted by a variety of methods, such as *in vitro* to *in vivo* extrapolation (IVIVE) or allometric scaling from preclinical species (Lu et al., 2006; Sodhi and Benet., 2021; Zou et al., 2012). In our experiments, allometric scaling from monkeys was used to predict the CL in humans. For macromolecular drugs, human CL is generally well-predicted with an average fold error of <2 using a fixed allometry exponent of 0.80; the interspecies scaling and prediction of human clearance is a comparison of small- and macromolecular drugs.

The predicted Kps values were also evaluated among the GastroPlus^TM^ build-in models, namely, Poulin and Theil-homogeneous, Poulin and Theil-extracellular, Berezhkovskiy, Rodgers-Leahy-Rowland, and Rodgers-single (Miller et al., 2019). Finally, the Rodgers-Leahy-Rowland model was selected for the final PBPK model. The final PBPK model also captured the C_max_ and AUC values in monkeys very well. After the preclinical model was validated, the respective *in vitro* human data were used to simulate the human plasma concentration versus time profiles of the MK-ASODN nanoliposomes at an IV dose of 90 mg, which was extrapolated from the monkey PK study, and estimated on the basis of biological exposure (US Food and Drug Administration, 2005). The predicted PK parameters of the MK-ASODN nanoliposomes using the human PBPK model showed moderate metabolism.

## 5 Conclusion

In summary, the MK-ASODN nanoliposomes demonstrated unique and favorable PK and disposition profiles following IV administration to monkeys and rats. The PK data of the MK-ASODN nanoliposomes were characterized by rapid distribution into tissues, resulting in the higherconcentration in the liver, the pharmacological target organ, and the kidney. The terminal t_1/2_ in plasma was clearly longer than that of the unmodified antisense nucleic acid drug. These favorable PK properties of the MK-ASODN nanoliposomes support further development in humans and serve as a model compound for the development of other drug candidates in this chemical class.

## Data Availability

The original contributions presented in the study are included in the article/Supplementary Material, further inquiries can be directed to the corresponding author.
