# Supplementary figures and images for: Pharmacokinetics and Disposition of Heparin-Binding Growth Factor Midkine Antisense Oligonucleotide Nanoliposomes in Experimental Animal Species and Prediction of Human Pharmacokinetics Using a Physiologically Based Pharmacokinetic Model
Source: Front Pharmacol. 2021 Nov 3;12:769538. doi: 10.3389/fphar.2021.769538 (PMC8595129; doi:10.3389/fphar.2021.769538)

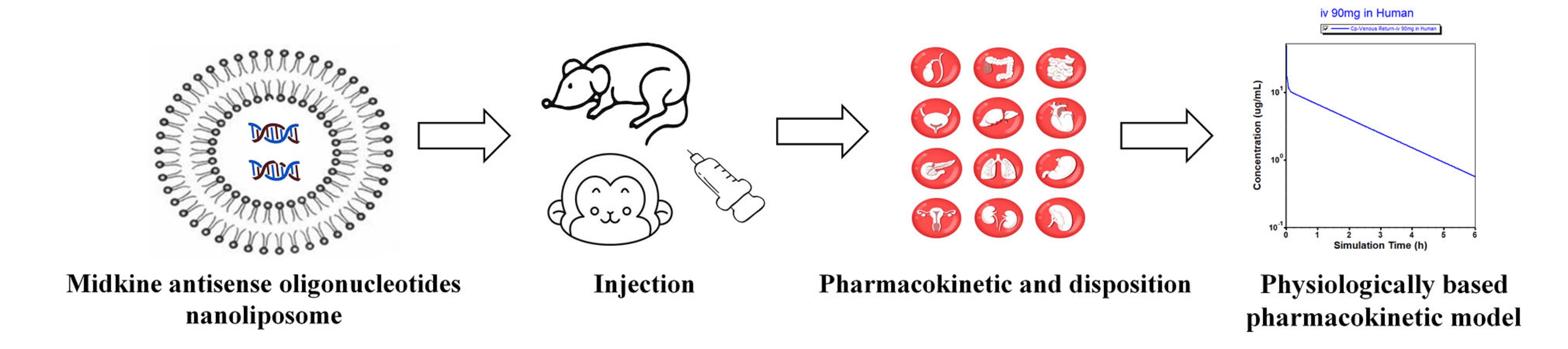

Supplement: Supplementary file 1 [file Image1.JPEG]

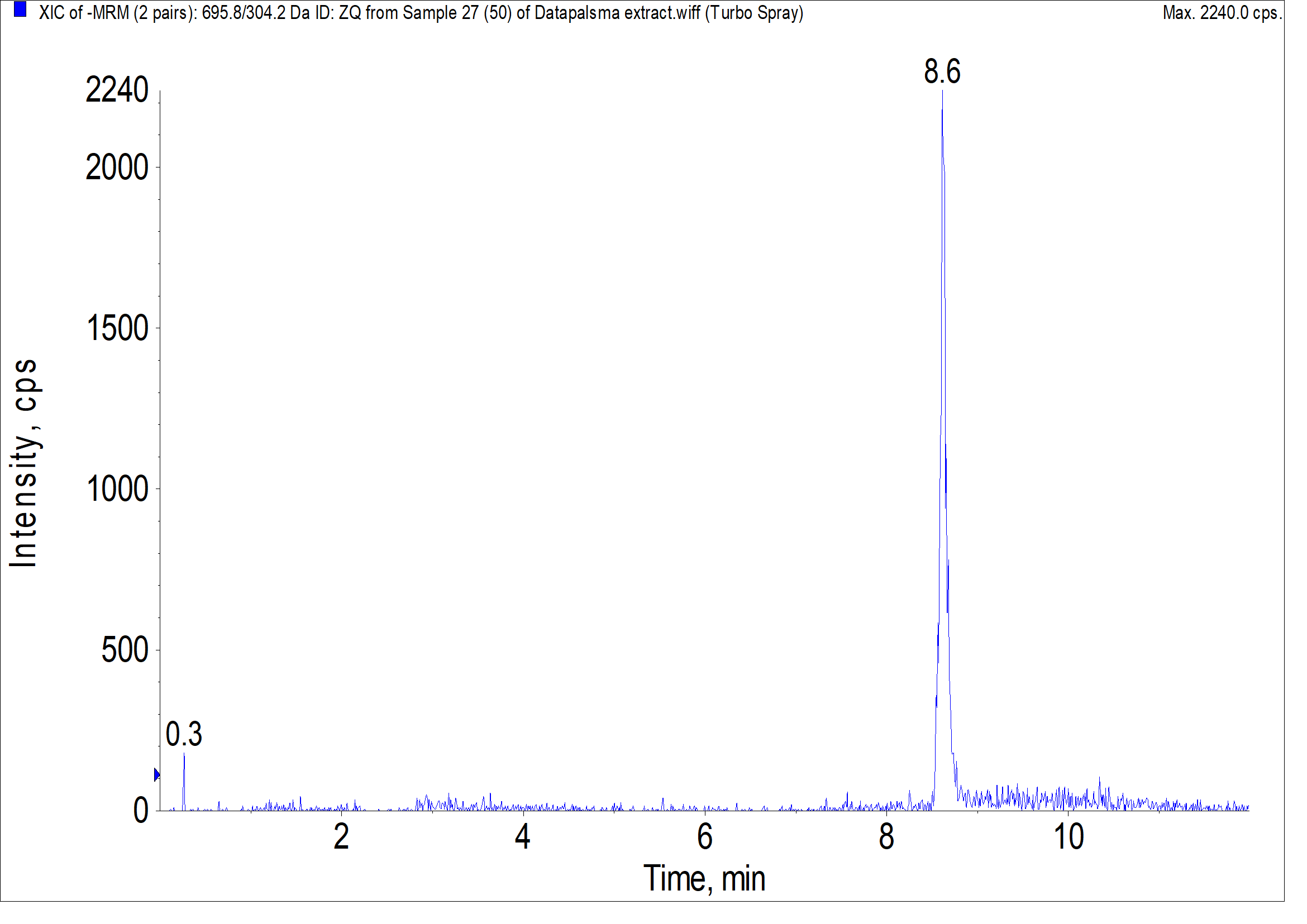

Supplement: Supplementary file 2 [file Image5.PNG]

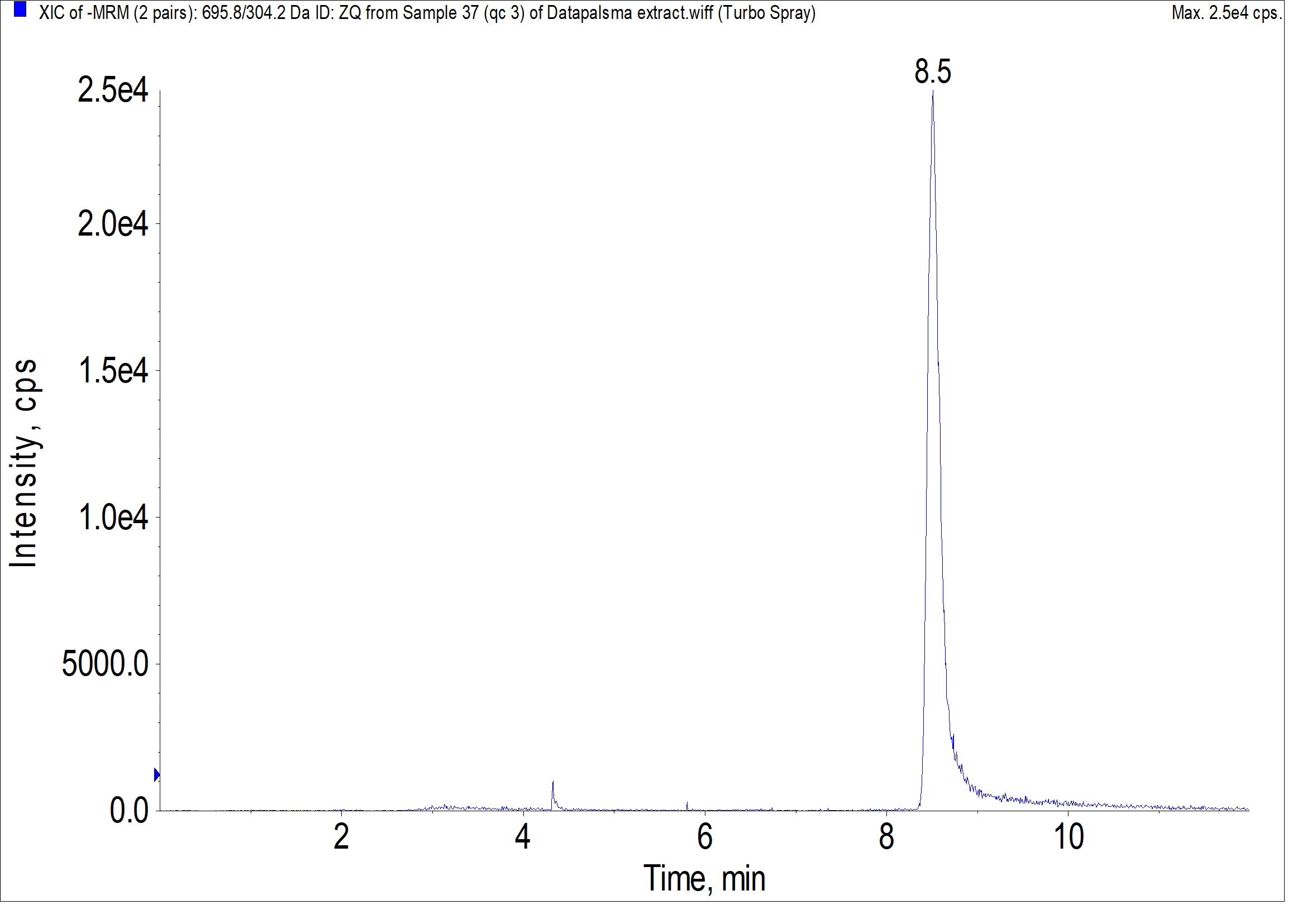

Supplement: Supplementary file 3 [file Image4.PNG]

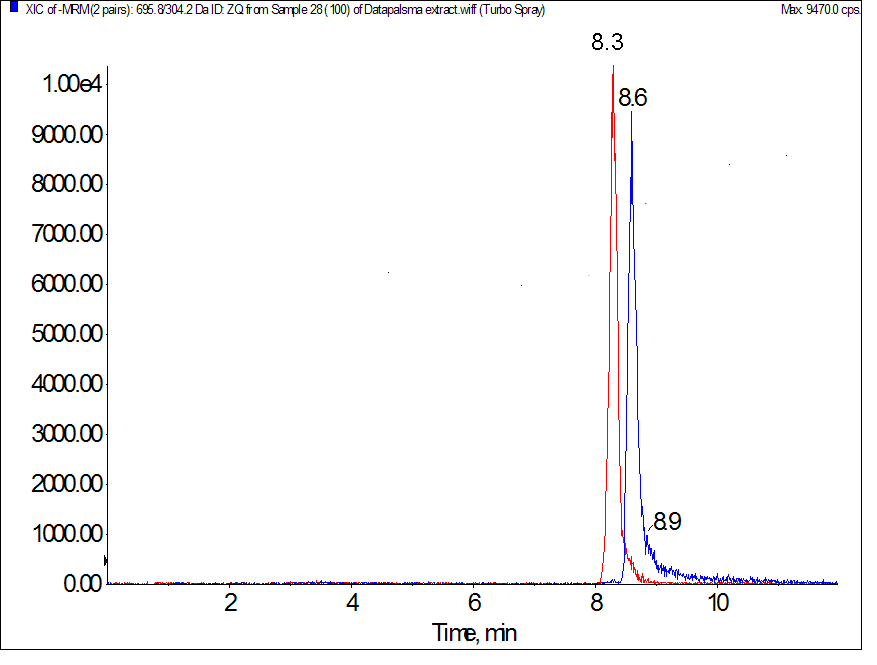

Supplement: Supplementary file 4 [file Image2.PNG]

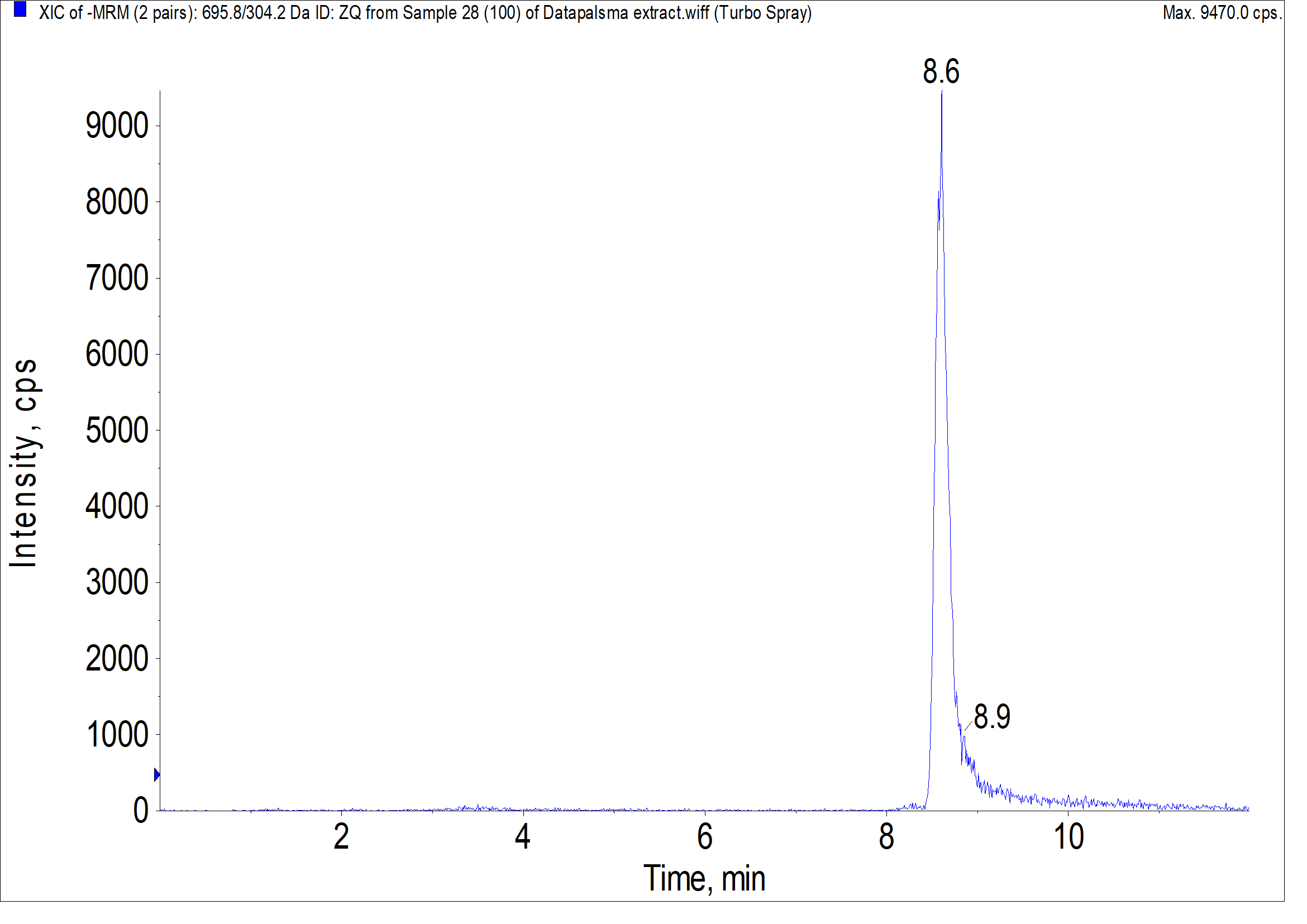

Supplement: Supplementary file 5 [file Image3.PNG]
